# Differences in residual volume above different tracheostomy tube cuffs depending on tube structure, tube tilt angle, and liquid viscosity

**DOI:** 10.1007/s00405-023-08281-6

**Published:** 2023-10-16

**Authors:** Takahiro Katsuno, Rumi Ueha, Aiko Fujisaki, Takeshi Unno, Carmel Cotaoco, Asako Kaneoka, Misaki Koyama, Taku Sato, Takao Goto, Kenji Kondo

**Affiliations:** 1 Department of Otolaryngology and Head and Neck Surgery, The University of Tokyo, Tokyo, Japan; 2 Swallowing Center, The University of Tokyo Hospital, The University of Tokyo, 7-3-1 Hongo Bunkyo-ku, Tokyo, 113-8655 Japan; 3 Ear Nose Throat Head and Neck Surgery Institute, The Medical City, Pasig, Philippines; 4 Rehabilitation Center, The University of Tokyo Hospital, Tokyo, Japan

**Keywords:** Tracheostomy tube, Cuff, Upper cuff residual volume, Suction port, Tube tilt angle, Liquid viscosity

## Abstract

**Introduction:**

Proper management of aspirated material above the tracheostomy tube cuff is crucial to prevent complications, such as aspiration pneumonia. This study aimed to thoroughly examine the effects of aspirated liquid viscosity, suction port positioning, and tube tilt angle on residual volume above the cuff (RVAC).

**Methods:**

Five types of tracheostomy tubes (approximately 9 mm outer diameter) were placed through a transparent cylinder with an inner diameter of 18 mm. The cuff was inflated to completely seal the interior of the cylinder. Four liquids with different viscosities were poured onto the cuff, and the liquid above the cuff was suctioned from the side port. The cylinder was angled at 90° and 20°, and each test was performed thrice to determine the average RVAC.

**Results:**

After side-port suctioning, some liquid residue was observed on the cuff of all tracheostomy tubes. The RVAC increased with higher liquid viscosity. The tubes with a longer distance from the suction port opening to the cuff top exhibited more RVAC. Moreover, the RVAC was almost the same regardless of the cylinder angle for tubes with a suction port on the lateral side. However, tubes with backside ports showed a decreased RVAC with cylinder tilt.

**Conclusions:**

This study underscores the persistence of residual material on cuffed tracheostomy tubes even with regular subglottic secretion drainage. This emphasizes the need for specialized tracheostomy tube development aimed at reducing post-suction RVAC. Improved designs can potentially minimize complications associated with residue accumulation.

## Introduction

Tracheostomy tube placement is a widely employed approach for airway management in patients with swallowing disorders. After tracheostomy is performed, a tracheostomy tube is inserted through the tracheostoma and managed [1]. In patients with dysphagia, aspirated material tends to accumulate above the cuff of the tracheostomy tube.

It is recommended to regularly remove the aspirated material that has accumulated at the upper cuff using the side suction port. Otherwise, it may flow into the trachea due to coughing or other stimuli or become a culture medium for bacterial growth due to stagnation [2]. Currently, many types of tracheostomy tubes with a suction port above the cuff are available to remove the sputum above the cuff. These are used for airway and respiratory management of patients with dysphagia [3]. Drainage of subglottic secretions, in which aspirated material at the upper cuff is suctioned from the side port of the tracheostomy tube, has been shown to reduce the incidence of ventilator-associated pneumonia in patients under ventilator management [3–5].

In our actual clinical experience, the amount of sputum above the cuff that can be suctioned varies depending on the viscosity of the sputum and changes in the body position, even in the same patient. Furthermore, after suctioning from the side port, medical professionals expect that there will be no residual material remaining above the cuff, but in actuality it is difficult to confirm how much residual material is left on the cuff. It is assumed that the position of the suction port on the tracheal tube, the patient’s position, and the viscosity of the aspirated material affect the residual volume above the cuff (RVAC); however, the relationships between these factors and RVAC have not been thoroughly examined.

This study aimed to examine RVAC after suction through the side port of cuffed tracheostomy tubes, taking into consideration the effects of the following factors: type of tracheal tube, viscosity of test boluses, suction port location, and tube tilt angle.

## Methods

### Material preparation

**Fig. 1:**
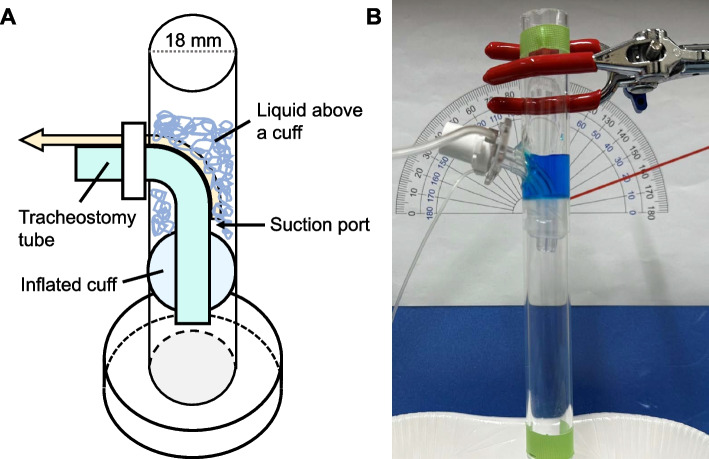
Experimental setup. **A** Schematic image of the experimental setup, **B** picture of the experimental setup. The blue-dyed liquid sits above the inflated cuff before suction

Five types of tracheostomy tubes with a suction port above the cuff were prepared for this study (Table 1). Tracheal tubes with an outer diameter of approximately 9 mm were used. A cuffed tracheal tube was placed through a hole in a transparent cylinder with an inner diameter of 18 mm, and the cuff was inflated (70 cm H_2_O) to completely seal off the inside of the cylinder (Fig. 1). To simulate the aspirated material/secretions in the experiment, water with four different viscosities (no thickening, mildly thick (100 mPa·s), moderately thick (250 mPa s), and extremely thick (400 mPa s)) [6] was used.

**Table 1:** Description of each cuffed tracheostomy tube

Tracheostomy tube	Sales company	Product name	Outer diameter (mm)	Inner diameter (mm)	Suction port location	Distance: suction port-cuff top (mm)	Suction port size (L × W mm)
Tube 1	Senko Medical Instrument Mfg. Co., Ltd. (Japan)	Sofit flex	9	6	Back side	3	5 × 5.5
Tube 2		Mera Sofit Clear	9.6	7	Left side	6	8.0 × 2.0
Tube 3	Smiths Medical Inc. (USA)	Blue Line Ultra^®^ (single-tube)	9.2	6	Left-to-back side	10	6.0 × 7.0
Tube 4	Koken Co., Ltd. (Japan)	Neo Breath (single-tube)	9	7	Back side	2	4.0 × 3.0
Tube 5		Meister Breath (single-tube)	9	6	Right side	2	3.0 × 3.0

### Experimental procedure

Liquids of different viscosities were then poured over the cuff one at a time. The upper cuff accumulation was suctioned from the side tube connecting the suction port until no more liquid came out. Because an endotracheal suction pressure of 10–20 kPa is recommended [7], a suction pressure of 20 kPa was applied. To calculate RVAC (ml), the difference between the weight of the entire device, including the residual fluid, and the weight of the device alone (g), was first calculated to obtain the weight of the residual on the cuff. This weight was then divided by the density of the liquid to obtain the volume (ml). Each test was performed three times, and the average residual volume was measured.

### Experimental items

**Fig. 2:**
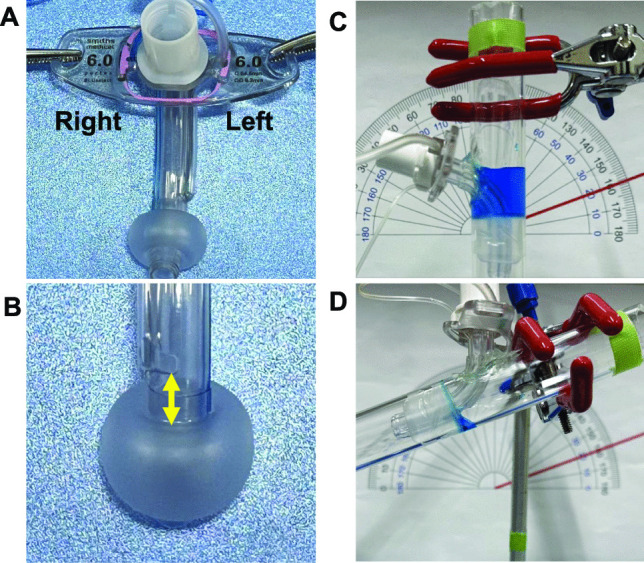
Evaluation methods. **A** Left and right sides of tracheal tube, **B** distance from the lower border of a suction port to the cuff top, **C** 90-degree cylinder angle, **D** 20-degree cylinder angle

1. Structural differences in various types of tracheal tubes
  To examine the effect of tracheostomy tube structure on RVAC, the following parameters were investigated: inner diameter, outer diameter, location of the suction port opening, distance from the cuff top to the lower border of the suction port opening, and size of the suction port opening for each tracheostomy tube used. The left and right sides of the tracheal tube were determined, as shown in Fig. 2A. The distance between the cuff and the suction port was defined as the distance from the top of the cuff to the lower end of the suction port opening when the cuff was sufficiently inflated (Fig. 2B).
2. Differences in RVAC depending on the liquid viscosity
  The effects of liquid viscosity on RVAC were determined for each tracheostomy tube.
3. Differences in RVAC depending on the suction port location
  The relationship between the suction port opening location (side or back) and RVAC, as well as the distance from the cuff to the suction port and RVAC, were investigated.
4. Effects of tracheostomy tube tilt angle on RVAC
  The experiments were performed three times, each with a cylinder at angles of 90° and 20° (Fig. 2C, D), and residual amounts (ml) at each angle were compared. The influence of the suction port location (side or back) on RVAC was investigated. An angle of 90° was set to simulate a patient in the sitting position, and an angle of 20° was set to simulate the position of the tracheostomy tube in a supine position using a pillow under the head. Realistically, no secretions accumulated above the cuff in a completely flat lying down position (0°). Therefore, this position was not simulated in this experiment.

## Results

### Structural details of various tracheostomy tubes

**Fig. 3:**
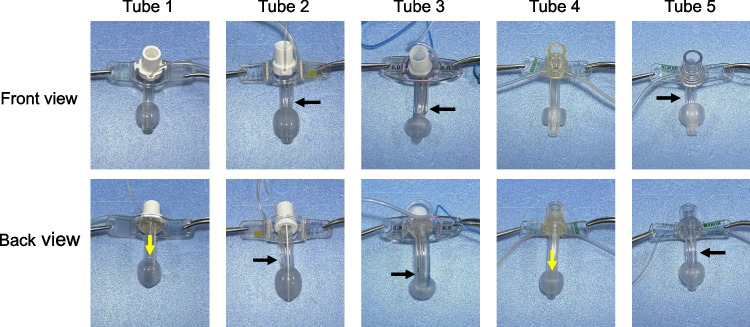
Structures of each tracheostomy tube. Yellow arrows indicate the dorsal suction ports. Black arrows point to the lateral suction ports

The structure of each tracheostomy tube is presented in Table 1 and Fig. 3. Regarding the position of the suction port, tracheostomy tubes 1 and 4 had suction ports on the back side, and tubes 2 and 5 had suction ports on the sides (one on the left and right). The tube 3 had a suction hole extending from the left lateral side to the back. The shortest distance from the top of the cuff to the lower edge of the suction port was 2 mm and the longest was 10 mm. The area of the suction ports varied from 9 to 42 mm^2^.

### Differences in RVAC depending on the liquid viscosity

**Fig. 4:**
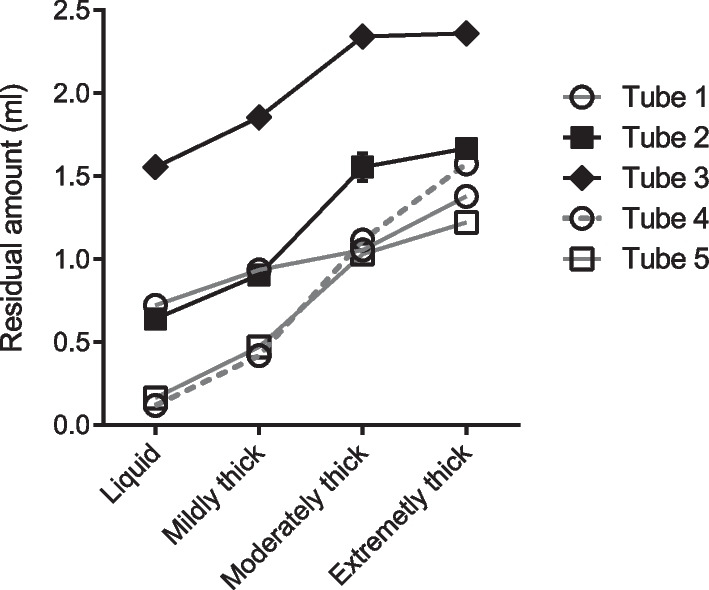
Differences in the upper cuff residual volume depending on the liquid viscosity in each tracheostomy tube. Black marks indicate tubes that have more distance from the cuff top to the suction port (tubes 2 and 3)

Using water with four different viscosities (no thickening, mildly thick, moderately thick, and extremely thick), the amount of residual material on the cuff after suctioning from the suction port was measured with the cylinder at a 90° angle. In all tubes, some amount of liquid remained on the cuff after suction through the suction port, even if the liquid had a low viscosity. Moreover, the residual volume increased as the viscosity of the liquid increased as well. Suction port size was not associated with an increased RVAC (Fig. 4).

### Differences in RVAC depending on the suction port location

RVAC at the 90-degree tube position was compared between tracheostomy tubes with the suction port located on the lateral side (tubes 2 and 5) and those with the suction port located on the back (tubes 1 and 4), but no specific trend was observed in the residual volume. Rather, the tubes with a longer distance from the suction port opening to the cuff top (tubes 2 and 3) had a larger amount of residual on the cuff (Fig. 4).

### Effects of tracheostomy tube tilt angle on upper cuff residual volume

**Fig. 5:**
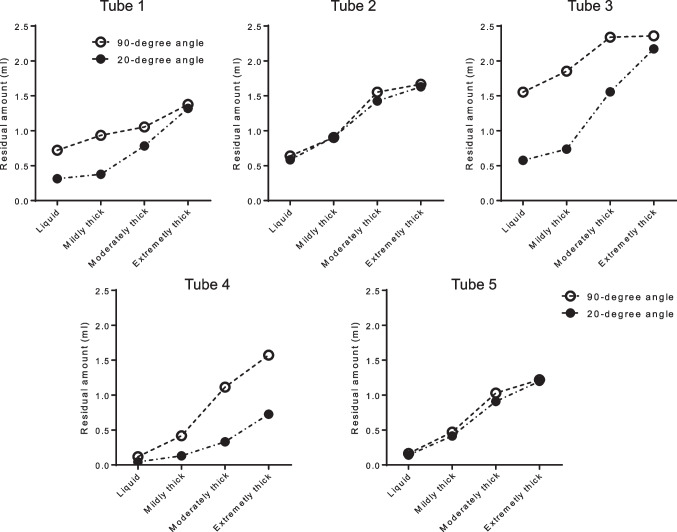
Differences in upper cuff residual volume depending on the tilt angle of tracheostomy tubes and liquid viscosity

For tubes 2 and 5, RVAC was almost the same whether the angle of the cylinder was at 90 degrees or 20 degrees, and no difference in residual volume was observed when the viscosity of the liquid was changed. In contrast, tubes 1, 3, and 4 had less RVAC at a 20-degree angle than at a 90-degree angle (Fig. 5). This means that the RVAC is almost the same regardless of the placement angle for tubes with a suction port on the lateral side, whereas for tubes with a suction port on the back side, the RVAC decreases when the cylinder is tilted.

However, even with the tubes with a suction port on the back side, the RVAC increased as the viscosity of the liquid increased. An extremely thick liquid left the greatest amount of RVAC in all tubes, but tube 4, which had the shortest distance between the suction port and cuff top, left the least amount of residue when the cylinder was tilted (Fig. 5).

## Discussion

In the present study, we demonstrated that some liquid was left on the upper cuff of the tube after side-port suctioning, regardless of the type of tracheostomy tube, and the higher the viscosity of the liquid, the more RVAC was observed. The longer the distance from the top of the tracheostomy tube cuff to the suction port opening, the greater is the RVAC. In addition, RVAC for tracheostomy tubes with suction ports on the sides remained almost constant regardless of the angle of the cylinder, while tubes with suction ports on the back showed less RVAC when the cylinder was tilted toward the supine position.

Secretions above the cuff of the endotracheal tube have been reported to be a significant factor in bacterial proliferation. When these secretions leak from around the cuff and migrate to the lungs, they can contribute to the development of pneumonia [8]. The drainage of subglottic secretions on the endotracheal or tracheostomy tube cuff has been recommended in recent years, as it reduces the risk of developing ventilator-associated pneumonia and postoperative pneumonia after cardiac surgery and contributes to a shorter intensive care unit stay [9–12]. This means that suctioning secretions above the cuff is effective in preventing aspiration of subglottic secretions into the lungs of patients with a tracheostomy tube. Consequently, this reduces RVAC and potentially prevents bacterial proliferation in the lower airway, with subsequent development of pneumonia. As demonstrated in the present study, in cases of highly viscous subglottic secretions, it is possible that a large amount of RVAC remains on the cuff after drainage through the suction port, presenting a potential challenge for infection control measures. To prevent subglottic secretions entering the lower airway, a proper fit between the cuff and the tracheal wall is essential. A larger cuff volume has the advantage of a larger area in contact with the trachea, effectively preventing aspiration of subglottic secretions. However, when using tracheostomy tubes with high-volume cuffs, it is preferable to manage them at appropriate pressures, as elevated cuff pressures may lead to decreased efficiency in swallowing and laryngeal elevation, along with an increased risk of tracheal mucosal injury [13].

Currently, various tracheostomy tubes are available. The most commonly used materials for the tube are polyvinyl chloride and silicone. Polyvinyl chloride softens at body temperature, making it easier to conform to the patient's anatomical structures. On the other hand, silicone is soft, temperature-resistant, resistant to bacterial colonization and biofilm formation, and can be sterilized [14]. The shape of tracheostomy tubes should ideally conform as much as possible to the anatomy of patients’ airway, and factors such as the inner and outer diameters, length, and curvature angle of the tubes should be chosen based on the anatomy of patients. In addition, in patients with excessive subglottic secretions, careful consideration should be given to the position of the suction port on the tubes. Based on the results of this study, patients who have a tracheostomy tube with a suction port opening at the back would benefit the most if suctioning is performed in a reclining position, as this demonstrated the least RVAC. Alternatively, for patients using a tracheostomy tube with a laterally positioned suction hole, RVAC may be reduced if suctioning is performed with the patient in a side-lying position with the suction port side-down. Regarding the size of the suction ports, further clinical research is required to determine whether a larger or smaller hole is more advantageous; a larger hole may facilitate easier drainage when the viscosity of the subglottic secretions is high.

The limitations of this study include differences in conditions between the experimental and clinical settings. In the present study, the cuff pressure was set higher than the clinical standard to prevent a gap between the cylinder and cuff, and a rigid transparent cylinder was employed as a substitute for the trachea, which did not suffer any deformation from suction pressure during side-port drainage. However, in the clinical setting, there is a warning about the potential risks associated with suction pressure, such as traction on the tracheal mucosa leading to blockage of the laterally situated suction holes or tracheal mucosal injury, as well as the risk of ulceration due to contact between the additional tube of the suction port and the tracheal mucosa [15].

As medical professionals, we need to understand that even after the drainage of subglottic secretions in patients with cuffed tracheostomy tubes, residual material may remain on the cuff; thus, continuous clinical monitoring and management are crucial. To reduce RVAC after suctioning, the development of a specialized tracheostomy tube is eagerly anticipated. Specifically, an optimal tracheostomy tube for patients requiring drainage of subglottic secretions should possess the following characteristics: a suction port positioned on the posterior side, considering the time spent in the supine position, and a short distance between the suction port opening and the cuff top.

## Conclusions

After side-port suctioning, some liquid residue was observed on the cuff of all tracheostomy tubes, regardless of tube type. Moreover, greater liquid viscosity and longer distances between the top of the tracheostomy tube cuff and the suction port led to increased RVAC. It is imperative for medical professionals to comprehend that despite drainage of subglottic secretions, residual material can persist on the cuff of cuffed tracheostomy tubes. Consequently, careful consideration is essential when choosing a tracheostomy tube, and should be based on the patient’s clinical condition.

## Data Availability

The data sets used and analyzed during the current study are available from the corresponding author upon reasonable request.
